# Improved survival of patients with hepatocellular carcinoma and disparities by age, race, and socioeconomic status by decade, 1983–2012

**DOI:** 10.18632/oncotarget.10930

**Published:** 2016-07-29

**Authors:** Shuncong Wang, Huanhuan Sun, Zhinan Xie, Jie Li, Guobin Hong, Dan Li, Saradhi Mallampati, Xiuling Zhou, Cuiling Zhou, Hongyu Zhang, Zhibin Cheng, Hong Shan, Haiqing Ma

**Affiliations:** ^1^ Department of Oncology, The Fifth Affiliated Hospital of Sun Yat-Sen University, Zhuhai, Guangdong 519000, China; ^2^ Department of Equipment Management, The Fifth Affiliated Hospital of Sun Yat-Sen University, Zhuhai, Guangdong 519000, China; ^3^ Department of Breast and Thyroid Surgery, The First Affiliated Hospital of Sun Yat-Sen University, Guangzhou, Guangdong 510080, China; ^4^ Department of Radiology, The Fifth Affiliated Hospital of Sun Yat-Sen University, Zhuhai, Guangdong 519000, China; ^5^ Center for Interventional Medicine, The Fifth Affiliated Hospital of Sun Yat-sen University, Zhuhai, 519000, China; ^6^ Guangdong Provincial Engineering Research Center for Molecular Imaging, Zhuhai, 519000, China; ^7^ Institute of Interventional Radiology, Sun Yat-sen University, Zhuhai, 519000, China; ^8^ Department of Laboratory Medicine and the Center for Stem Cell and Developmental Biology, The University of Texas MD Anderson Cancer Center, Houston, TX 77030, USA

**Keywords:** hepatocellular carcinoma, incidence, relative survival, SEER, socioeconomic status

## Abstract

Hepatocellular carcinoma (HCC), accounting for the majority of liver cancer, is a highly aggressive malignancy with poor prognosis and therefore adds up the financial burden. Incidence data of HCC in three decades during 1983-2012 were extracted from the Surveillance, Epidemiology, and End Results (SEER) database with incidence rates of 1.9, 3.1 and 4.9 per 100,000 respectively. In addition, to evaluate the survival changes in the same period, a total of 63,640 HCC cancer cases were accessed from SEER database. The six-month relative survival rates improved each decade from 31.0% to 42.9% to 57.2% and the higher increase can be seen in the last two decades. More importantly, the disparities of survival among different racial groups and socioeconomic status (SES) were confirmed by the inferiority of survival in Black race and high-poverty group respectively. This research analyzed the incidence and survival data of HCC in the past three decades and may help predict the future trends of incidence and survival. Furthermore, this study may help better design healthcare policies and clinical management programs to balance the disparities of survival between SES groups, races, ages and sexes confirmed in this study and thereby improve the clinical management of HCC.

## INTRODUCTION

The incidence of hepatocellular carcinoma (HCC) in the United States has doubled in the past twenty years, and 39,230 cases of liver cancer were estimated in 2016 [1]. The long-term prognosis for HCC is poor with 5-year survival rate of approximately 5-6% [2, 3]. In the era of precision medicine and translational medicine, there is an urgent need not only to clarify pathogenesis but also to analyze clinical data, which could not only help improve the treatment of HCC but help politicians to balance the disparities among patients from different races and sociodemographic status (SES) background when drafting new medical policies.

According to the estimation of American Cancer Society, there will be approximately 39,230 new liver cancer cases, about three fourths of which will be HCC, and 27,170 deaths from liver cancer in 2016. In addition, the prognosis for HCC remained poor, despite improvement in HCC treatment such as application of Sorafenib, radiofrequency ablation (RFA) and novel agents for transcatheter arterial chemoembolization (TACE). However, previous publications studying the prognosis of HCC were not comprehensive enough, for some mainly focused on specific patients from certain regions, some focusing on patients received specific treatments, and some only clarified the effects of sex, SES, race, AJCC stage or marriage status on prognosis [4–9].

Recently accumulating evidences showed that the racial and SES heterogeneities became increasingly significant in US health care system and it has drawn increasing attention from health care providers and politicians. Therefore this study was aimed not only to demonstrate the changes in relative survival rates (RSRs) in HCC patients from 1983 to 2012, but also to determine whether age, sex, race and SES could affect the RSRs by analyzing data from the Surveillance, Epidemiology, and End Results (SEER) database.

## RESULTS

### Changes in HCC incidence between 1983 and 2012

**Figure 1:**
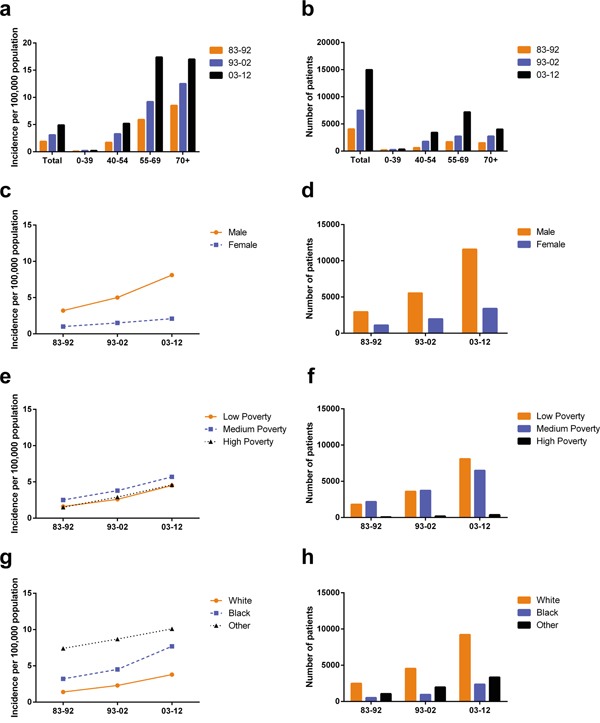
Summary incidences of patients diagnosed with HCC between 1983 and 2012 at the original nine SEER sites Incidence **a.** and number **b.** of HCC cases are shown by age group (total and ages 0–39, 40–54, 55–69 and 70+ years) and calendar period. Incidence **c, e, g.** and number **d, f, h.** of HCC cases are grouped by sex, SES, and race, respectively.

In order to keep consistence of the registry sites surveyed across three decades and thereby improve incidence comparability across three decades, we collected incidence data from the original nine registry sites in SEER database. Totally 26535 HCC cases were identified between 1983 and 2012. The total incidence of HCC during 1983-1992 was lower (1.9 per 100,000) than that during 1993-2002 (3.1 per 100,000) which was also lower than that during 2003-2012 (4.9 per 100,000) (Figure 1 and Supplementary Table S1). And the similar tendency can be found in each age group. In the first two decades, the highest incidence rates were found in age group over 70 with 8.5 per 100,000 and 12.5 per 100,000 respectively. However, in the third decade the highest incidence rate was shown in age group 55-69 with 17.4 per 100,000 which was slightly higher than that of age group over 70 (17.0 per 100,000). However, the absolute number of HCC patients increased in all age groups as the general population grew (Figure 1b, Supplementary Table S1.). The HCC incidence per 100,000 increased markedly in the age groups between 55-69 and over 70, from 5.9 in 1983–1992 to 9.2 in 1993–2002 to 17.4 in 2003–2012 and from 8.5 in 1983–1992 to 12.5 in 1993–2002 to 17.0 in 2003–2012 respectively (Figure 1a, Supplementary Table S1.). In addition, higher incidence and patient number per 100,000 were shown in males compared with females (3.2 vs. 1.0 in 1983–1992, 5.0 vs. 1.5 in 1993–2002, and 8.1 vs. 2.1 in 2003–2012; Figure 1c, 1d, Supplementary Table S1.).

### HCC incidence in different SES groups and races

The incidence rates across three SES groups increased in three decades with the highest incidence in the medium poverty group (from 2.5 in 1983-1992 to 3.8 in 1993-2002 to 5.7 in 2003-2012). In addition, the low-poverty group and the high-poverty group shared similar HCC incidence, from 1.6 in 1983-1992 to 2.6 in 1993-2002 to 4.5 in 2003-2012 and from 1.5 in 1983-1992 to 2.9 in 1993-2002 to 4.6 in 2003-2012 respectively. Whites showed the lowest HCC incidence during the three decades and Others showed the highest (Figure 1e, 1f and Supplementary Table S1). However, because the incidence of Blacks increased more rapidly in the last two decades, the incidence difference between Others and Blacks narrowed whereas the incidence gap between Whites and Blacks started to widen in the last two decades.

### Relative survival estimates for the eighteen SEER sites between 1983 and 2012

**Figure 2:**
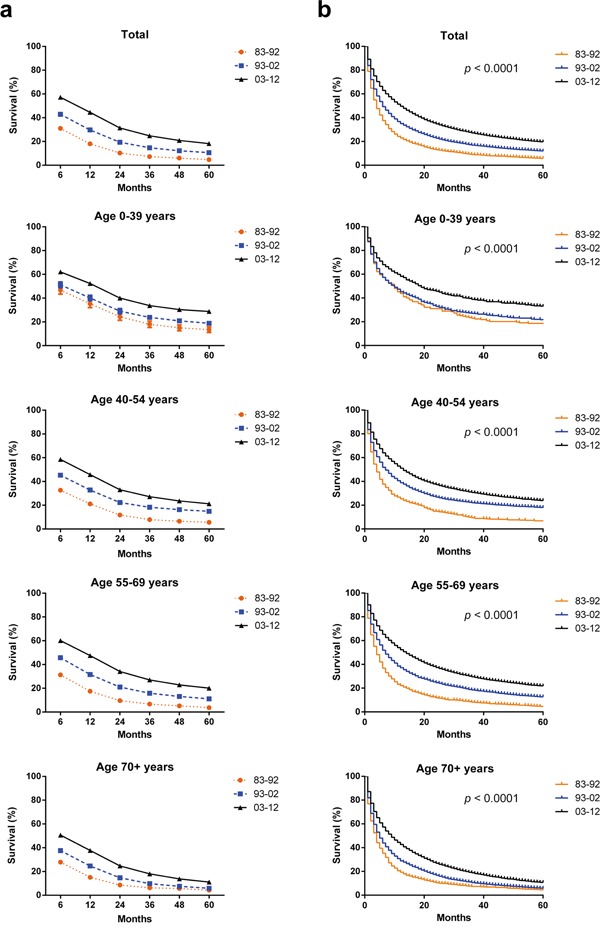
Trends in 5-year relative survival rates **a.** and Kaplan-Meier survival analysis **b.** for patients with HCC at eighteen SEER sites in 1983-1992 (black), 1993-2002 (blue) and 2003-2012 (orange) respectively according to age group (total and ages 0–39, 40–54, 55–69, and 70+ years).

We have identified 63640 HCC cases across three decades from eighteen registry sites with median survival of 4 months, 6 months and 12 months in each decade respectively. Both RSR and survival time improved each decade in patients with HCC in all age groups (Table 1 and Figure 2a). The 6-month RSRs improved from 31.0% to 42.9% to 57.2% each decade, with 27.7% increase in the first two decades and 33.3% in the last two decades. The increasing tendency of survival time was obtained after up to 5 years of follow-up. Similarly Kaplan-Meier survival analysis also confirmed the improvement of survival time in all age groups in three decades (Figure 2b). However the long-term survival seemed gloomy with 5-year RSRs of 4.7%, 10.6% and 18.2% respectively in three decades.

**Table 1:** Relative survival rates of hepatocellular carcinoma patients during the periods of 1983-1992, 1993-2002, and 2003-2012 at eighteen SEER sites

Age Group	Decade		
	1983-1992	1993-2002	2003-2012
6-Mo RSR			
All	31.0 ± 0.7 (4333)	42.9 ± 0.4(15805)***	57.2 ± 0.2(43502)***
0-39	46.8 ± 3.4(211)	51.2 ± 2.3 (505)***	62.0 ± 1.7(811)***
40-54	32.6 ± 1.8(674)	45.3 ± 0.8(3801)***	58.5 ± 0.5(10031)***
55-69	31.3 ± 1.1(1825)	45.7 ± 0.7(5890)***	60.1 ± 0.3(20763)***
70+	27.9 ± 1.1(1623)	37.5 ± 0.7(5599)***	50.6 ± 0.5(11897)***
12-Mo RSR			
All	18.0 ± 0.6	29.7 ± 0.4***	44.5 ± 0.3***
0-39	35.3 ± 3.3	40.1 ± 2.2	52.3 ± 1.8***
40-54	21.1 ± 1.6	32.8 ± 0.8***	45.8 ± 0.5***
55-69	17.5 ± 0.9	31.6 ± 0.6***	47.5 ± 0.4***
70+	15.1 ± 0.9	24.6 ± 0.6***	37.7 ± 0.5***
24-Mo RSR			
All	10.3± 0.5	19.4 ± 0.3***	31.4 ± 0.2***
0-39	24.4 ± 3.0	29.0 ± 2.1	40.0 ± 1.8***
40-54	11.8 ± 1.3	22.3 ± 0.7***	33.0 ± 0.5***
55-69	9.6 ± 0.7	21.0 ± 0.5***	34.1 ± 0.4***
70+	8.6 ± 0.7	14.7 ± 0.5***	24.7 ± 0.5***
36-Mo RSR			
All	7.3 ± 0.4	14.7 ± 0.3***	24.8 ± 0.2***
0-39	18.0 ± 2.7	23.8 ± 1.9	33.7 ± 1.8**
40-54	7.9 ± 1.1	18.3 ± 0.6***	27.2 ± 0.5***
55-69	6.7 ± 0.6	15.9 ± 0.5***	27.0 ± 0.4***
70+	6.3 ± 0.7	9.8 ± 0.4***	18.0 ± 0.4***
60-Mo RSR			
All	4.7 ± 0.4	10.6 ± 0.3***	18.2 ± 0.2***
0-39	13.5 ± 2.4	18.9 ± 1.8	28.8 ± 1.9**
40-54	5.6 ± 0.9	14.9 ± 0.6***	21.2 ± 0.5***
55-69	3.7 ± 0.5	11.1 ± 0.4***	20.1 ± 0.4***
70+	4.3 ± 0.6	5.9 ± 0.4	11.1 ± 0.4***

Abbreviations: Mo, month; RSR, relative survival rate; SEM, standard error of the mean.

* *p* < 0.01,

** *p* < 0.001, and

*** *p* < 0.0001 for comparisons with the preceding decade.

**Figure 3:**
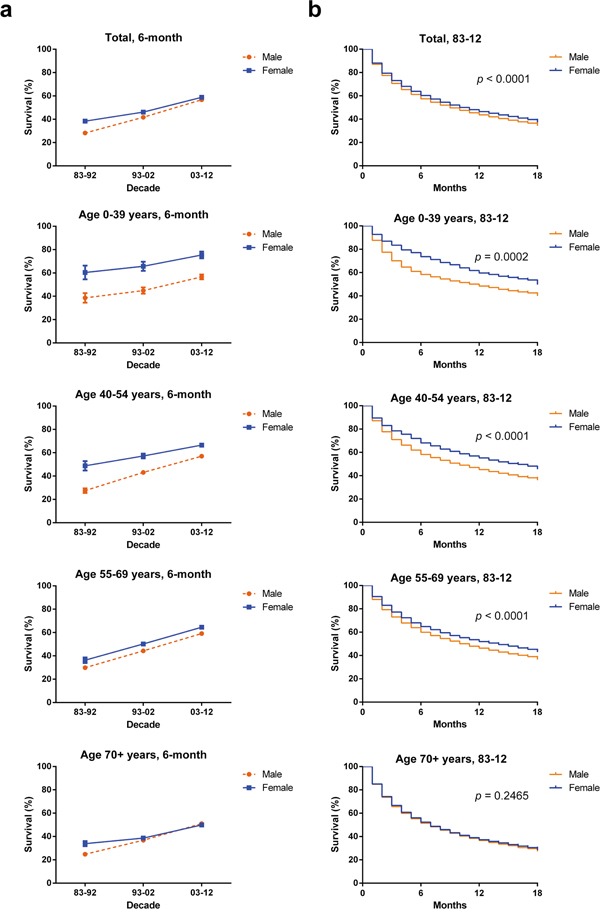
Six-month relative survival rates from 1983 to 2012 **a.** and Kaplan-Meier survival analysis from 1983 to 2012 **b.** for male (orange) and female (blue) with HCC at eighteen SEER sites by age group (total and ages 0–39, 40–54, 55–69, and 70+ years).

Survival improvement across three decades can be seen in both sexes (Table 2 and Figure 3a). In the first decade, survival superiority can be found in female with higher 6-month RSRs (38.4% vs. 28.2%, *p* < 0.0001), however this survival superiority in female diminished in the last two decades (46.1 vs. 41.7% in 1993–2002 and 58.8% vs. 56.7% in 2003–2012; Table 2). As demonstrated in Supplementary Table S2 and Supplementary Figure S1, similar trends were shown in 12-month and 24-month RSRs. Significant survival improvements across three decades can be observed in the any age group and total population according to Kaplan-Meier survival analysis (*p* < 0.0001) (Supplementary Figure S2).

**Table 2:** Six-month relative survival rates of HCC patients according to sex, age group, and calendar period from 1983 to 2012 at eighteen SEER sites. Data are means ± standard error of the mean, with number of patients in parentheses

Decade	Age Group	Sex	
		Male	Female
83-92	6-Mo RSR		
	All	28.2 ± 0.8(3145)	38.4 ± 1.4(1188)***
	0-39	38.6 ± 4.1(144)	60.40 ± 5.9(67)
	40-54	27.5 ± 2.0(514)	48.8 ± 4.0(160)***
	55-69	29.9 ± 1.2 (1416)	36.3 ± 2.4(409)
	70+	24.8 ± 1.3(1071)	33.9 ± 2.1 (552)**
93-02	6-Mo RSR		
	All	41.7 ± 0.5(11637)	46.1 ± 0.8(4168)***
	0-39	44.9 ± 2.7(255)	65.7 ± 3.9(150)***
	40-54	43.1 ± 0.9(3224)	57.2 ± 2.0(587)***
	55-69	44.3 ± 0.7(4493)	50.2 ± 1.3(1397)***
	70+	36.8 ± 0.8 (3565)	38.6 ± 1.1 (2034)
03-12	6-Mo RSR		
	All	56.7 ± 0.3(33662)	58.8 ± 0.5(9840)**
	0-39	56.6 ± 2.1(584)	75.4 ± 2.9(227)***
	40-54	57 ± 0.5 (8506)	66.5 ± 1.2(1525)***
	55-69	59.1 ± 0.4(16911)	64.5 ± 0.8(3852)***
	70+	51.0 ± 0.6 (7661)	49.9 ± 0.8 (4236)

Abbreviations: mo, month; RSR, relative survival rate; SEM, standard error of the mean.

* *p*< 0.01,

** *p*< 0.001, and

*** *p*< 0.0001 for comparisons with the Male group.

The RSRs gap between males and females was more pronounced in some age groups (Table 2 and Figure 3a). For example, females aged 0–39 years had a significantly higher 6-month RSRs compared with male counterparts in each decade (60.4% vs. 38.6% in 1983–1992, 65.7% vs. 44.9% in 1993–2002, 75.4% vs. 56.6% in 2003–2012; *p* < 0.0001 for each). And similar 6-month relative survival superiority can also be seen in females aged 40-54 years compared with male counterparts (48.8% vs. 27.5% in 1983–1992, 57.2% vs. 43.1% in 1993–2002, 66.5% vs. 57% in 2003–2012; *p* < 0.0001 for each). However the survival difference between sexes was smaller in age group 55-69 than in age group 0-39. And the survival gap was narrower in the 70+ age group in which the 6-month RSRs gap between sexes narrowed first and then even reversed in time order. The difference in 12-month and 24-month RSRs between sexes kept narrowing with increasing age (supplementary Table S2 and supplementary Figure S3).

The age-dependent survival difference between sexes was illustrated by Kaplan-Meier curves (Figure 3b). Survival advantage in females can only be seen in the age groups younger than 70 years (with *p* = 0.0002 for group aged 0-39; with *p* < 0.0001 for group aged 40-54; with *p* < 0.0001 for group aged 55-69 respectively) but not in the age group 70+ (*p* = 0.2465).

In addition, Cox regression analysis during three decades demonstrated that sex, age, race and SES were independent predictors for prognosis. Hazard ratios for age, race and SES were greater than 1 indicating that older age, Black race and med-high-poverty were related with shorter survival. However hazard ratio for sex was less than 1 showing that female population were associated with longer survival time. However, when the patients were divided by different decades, sex, age and race were significantly related with the survival time across three decades, whereas SES became significantly related with survival time only in the last two decades with *p* = 0.004 and *p* < 0.001 respectively (Table 3). After stratification of patients by age, race was associated with overall survival in all age groups. Age was significantly related with the survival time in most age groups except age group 40-54 (*p* = 0.522) and sex and SES were independent predictors in most age groups except age group 70+ (*p* = 0.022 and *p* = 0.010) (Supplementary Table S3).

**Table 3:** Summary data for Cox regression analysis of survival in patients with HCC in each decade from 1983 to 2012 at eighteen SEER sites

Variable	Hazard Ratio (95% CI)	*p*-value
**All 1983-2012**		
Univariate		
Sex	0.941 (0.919 − 0.963)	< 0.001
Age	1.016 (1.015 − 1.017)	< 0.001
Race	1.151 (1.121 − 1.181)	< 0.001
SES	1.086 (1.064 − 1.108)	< 0.001
Multivariate		
Sex	0.878 (0.857 − 0.898)	< 0.001
Age	1.017 (1.016 − 1.018)	< 0.001
Race	1.204 (1.173 − 1.236)	< 0.001
SES	1.081 (1.059 − 1.104)	< 0.001
**All 1983-1992**		
Univariate		
Sex	0.775 (0.717 − 0.838)	< 0.001
Age	1.013 (1.010 −1.015)	< 0.001
Race	1.148 (1.046 − 1.260)	= 0.004
SES	1.082 (1.009 − 1.160)	= 0.027
Multivariate		
Sex	0.772 (0.714 − 0.835)	< 0.001
Age	1.014 (1.011 − 1.016)	< 0.001
Race	1.195 (1.089 − 1.312)	< 0.001
**All 1993-2002**		
Univariate		
Sex	0.923 (0.885 − 0.963)	< 0.001
Age	1.014 (1.012 − 1.015)	< 0.001
Race	1.167 (1.109 − 1.228)	< 0.001
SES	1.053 (1.013 − 1.095)	= 0.010
Multivariate		
Sex	0.866 (0.830 − 0.904)	< 0.001
Age	1.015 (1.014 − 1.017)	< 0.001
Race	1.246 (1.183 − 1.312)	< 0.001
**All 2003-2012**		
Univariate		
Sex	0.928 (0.901 − 0.956)	< 0.001
Age	1.016 (1.014 − 1.017)	< 0.001
Race	1.157 (1.121 − 1.194)	< 0.001
SES	1.154 (1.124 − 1.184)	< 0.001
Multivariate		
Sex	0.865 (0.840 − 0.891)	< 0.001
Age	1.017 (1.016 − 1.018)	< 0.001
Race	1.198 (1.160 − 1.237)	< 0.001
SES	1.150 (1.120 − 1.180)	< 0.001

Abbreviations: 95% CI, 95% confidence interval; SES, socioeconomic status.

### HCC survival in different races and SES groups

**Figure 4:**
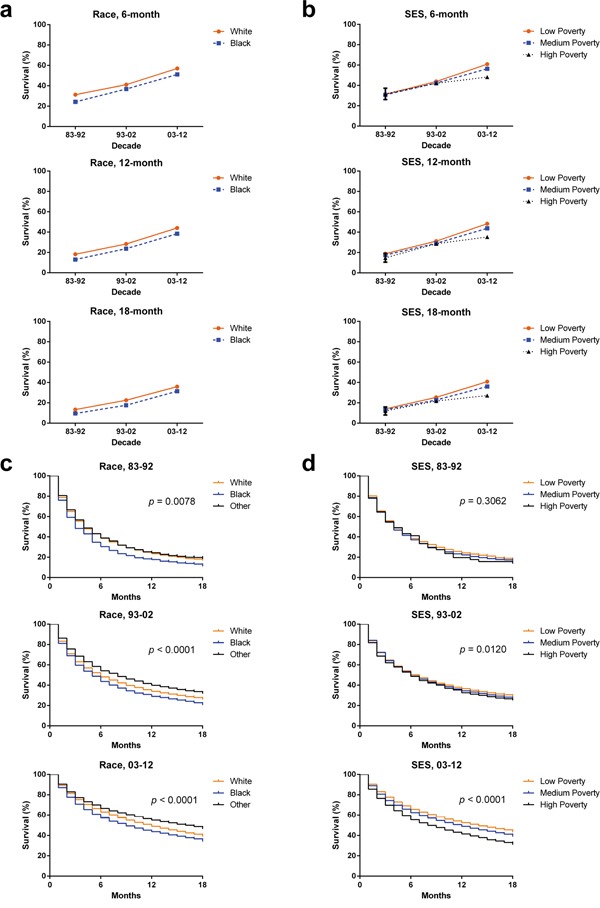
Six-month, 12-month, and 18-month relative survival rates according to race **a.** and SES/county-level poverty rates **b.** and Kaplan-Meier survival analysis according to races **c.** including White (orange), Black (blue) and Others (black) and SES/county-level poverty rates **d.** in low-poverty (orange), medium poverty (blue) and high poverty (black) for patients with HCC at eighteen SEER sites from 1983 to 2012.

Whites had higher 6-month RSRs compared with Blacks in the first decade (31.3% vs. 24.2%; *p*= 0.011, Figure 4a and Table 4) and survival time of Whites and Blacks was significantly differed in the second decade (41.1% vs. 36.9%; *p*= 0.0010). In addition, the survival gap between Whites and Blacks became even wider in the third decade (57.0% vs. 51.1%; *p*< 0.0001). The widening tendency in time order was also seen in 12-month and 24-month RSRs (Supplementary Table S4). The survival trend of patients immigrated from Asian can be reflected by that of Others due to the fact that Asian patients accounted for approximately 89.2% of Others.

**Table 4:** Six-month relative survival rates of HCC patients according to race, age group, and calendar period from 1983 to 2012 at eighteen SEER sites

Decade	Age Group	Race		
		White	Black	Other
83-92	6-Mo RSR			
	All	31.3 ± 0.9 (2638)	24.2 ± 1.9 (534)**	33.3 ± 1.4 (1155)
	0-39	64.8 ± 4.9 (97)	32.3 ± 8.4 (31)**	31.3 ± 5.1 (83)***
	40-54	34.7 ± 2.7 (306)	25.4 ± 4.1 (111)	32.7 ± 3.0 (255)
	55-69	30.9 ± 1.4 (1112)	24.1 ± 2.8 (232)	36.1 ± 2.2 (478)
	70+	27.9 ± 1.4 (1123)	22.0 ± 3.3 (160)	30.4 ± 2.5 (339)
93-02	6-Mo RSR			
	All	41.1 ± 0.5 (9765)	36.9 ± 1.2 (1806)**	49.3 ± 0.8 (4183)***
	0-39	62.9 ± 3.3 (221)	40.0 ± 5.8 (74)**	42.3 ± 3.5 (207)***
	40-54	46.3 ± 1.1 (2196)	36.1 ± 2.0 (612)***	48.6 ± 1.6 (988)
	55-69	43.9 ± 0.8 (3493)	39.6 ± 1.9 (704)	51.6 ± 1.2 (1670)***
	70+	34.3 ± 0.8 (3855)	32.8 ± 2.4 (416)	47.8 ± 1.4 (1318)***
03-12	6-Mo RSR			
	All	57.0 ± 0.3 (29121)	51.1 ± 0.7 (5840)***	61.8 ± 0.5 (8348)***
	0-39	71.6 ± 2.4 (388)	55.0 ± 4.1 (156)**	52.2 ± 3.1 (263)***
	40-54	59.7 ± 0.6 (6725)	49.2 ± 1.3 (1448)***	61.1 ± 1.2 (1816)
	55-69	60.5 ± 0.4 (13848)	53.3 ± 0.9 (3361)***	64.9 ± 0.8 (3448)***
	70+	48.1 ± 0.6 (8160)	45.4 ± 1.8 (875)	59.4 ± 1.0 (2821)***

Data are means ± standard error of the mean, with number of patients in parentheses.

Abbreviations: Mo, month; RSR, relative survival rate; SEM, standard error of the mean.

* *p* < 0.01,

** *p* < 0.001, and

*** *p* < 0.0001 for comparisons with the White group.

Low-poverty group had the highest 6-month RSRs whereas the lowest RSRs were shown in the high-poverty group and similar disparities can be found in 12-month and 24-month RSRs (Figure 4b, Table 5 and Supplementary Table S5). Although all SES groups showed survival improvements across three decades, they showed significantly different RSRs in the third decade. For example, the low-poverty, medium-poverty, and high-poverty groups in 1983–1992 shared similar 6-month survival rates with 31.3%, 30.7%, and 31.7%, respectively. However, statistically significant difference in 6-month survival rate can be seen among three SES groups in 2003-2012 (60.9%, 56.4%, and 48.2% in the low-poverty, medium-poverty, and high-poverty groups, respectively; *p*< 0.0001 Table 5). Similar widening tendency of survival gap among different SES groups was observed in the 12-month and 24-month RSRs (Supplementary Table S5).

**Table 5:** Six-month relative survival rates of HCC patients according to SES, age group, and calendar period from 1983 to 2012 at eighteen SEER sites

Decade	Age Group	SES		
		Low Poverty	Medium Poverty	High Poverty
83-92	6-Mo RSR			
	All	31.3 ± 1.1 (1827)	30.7 ± 0.9 (2435)	31.7 ± 5.6 (71)
	0-39	53.8 ± 5.6 (80)	42.4 ± 4.3 (131)	0.0 ± 0.0 (0)
	40-54	36.0 ± 3.0(266)	30.6 ± 2.3 (403)	20.0 ± 17.9 (5)
	55-69	30.7 ± 1.7 (754)	32.0 ± 1.5 (1042)	24.3 ± 8.0 (29)
	70+	27.7 ± 1.7 (727)	27.5 ± 1.6 (859)	39.1 ± 8.2 (37)
93-02	6-Mo RSR			
	All	43.9 ± 0.7 (5236)	42.4 ± 0.5 (9772)	42.1 ± 1.8 (795)
	0-39	60.4 ± 3.7 (181)	45.2 ± 2.9 (302)	57.9 ± 10.7 (22)
	40-54	48.4 ± 1.4 (1232)	43.8 ± 1.0 (2360)	43.2 ± 3.4 (218)
	55-69	48.0 ± 1.2 (1881)	44.6 ± 0.8 (3741)	45.1 ± 3.1 (268)
	70+	35.5 ± 1.1 (1942)	38.6 ± 0.9 (3369)	37.2 ± 2.9 (287)
03-12	6-Mo RSR			
	All	60.9 ± 0.4 (14229)	56.4 ± 0.3 (25673)***	48.2 ± 0.9 (3594)***
	0-39	64.9 ± 2.9 (279)	61.4 ± 2.3 (457)	54.1 ± 5.9 (74)
	40-54	63.5 ± 0.9 (3206)	57.3 ± 0.7 (5882)***	48.4 ± 1.6 (941)***
	55-69	64.5 ± 0.6 (6677)	59.1 ± 0.5 (12408)***	49.7 ± 1.3 (1676)***
	70+	52.5 ± 0.8 (4067)	50.3 ± 0.6 (6926)	44.7 ± 1.7 (903)***

Data are means ± standard error of the mean, with number of patients in parentheses.

Abbreviations: Mo, month; RSR, relative survival rate; SEM, standard error of the mean.

* *p* < 0.01,

** *p* < 0.001, and

*** *p* < 0.0001 for comparisons with the Low Poverty group.

Interestingly, Whites and Blacks had different SES distribution with most black patients being defined as medium-poverty compared with white counterparts (68.5% vs. 56.1%) whereas most white patients being defined as low-poverty compared with black counterparts (36.2% vs. 21.1%; Supplementary Figure S4, Supplementary Table S6). The correlation between the variable race and the variable SES was illustrated by Spearman's rank correlation coefficient of 0.118 with *p* < 0.001. In addition, the survival difference between Whites and Blacks reflected the survival gap between low-poverty group and medium-poverty group. As demonstrated in Figure 4c, survival gap between Whites and Blacks widened and became significant in the last two decades (*p* < 0.0001). And similarly widening survival gap among three SES groups was confirmed with decreasing *p* values (Figure 4d).

## DISCUSSION

In general, we demonstrated that both incidence and median survival of HCC increased in each decade during 1983-2012 and a more significant increase can be seen in the last two decades. However, the long-term prognosis remains relatively poor with 5-year RSR of 18.2% during 2003-2012.

We found that overall HCC incidence per 100,000 kept increasing over three decades. Risk factors for HCC cover a broad spectrum conditions including HBV infection, HCV infection, metabolic syndrome, non-alcoholic fatty liver disease (NAFLD) and cirrhosis [10, 11]. The increasing incidence of HCC may be attributed to the rising prevalence of obesity and type 2 diabetes mellitus (T2DM)-the two major risk factors of NAFLD [12]. However, things are expected to be better in the future. With the development in the prevention of HCV spreading in the USA, the prevalence of HCV per 100,000 has decreased, from approximately 7.4 in 1982–1989 to 0.7 in 1994–2006 to approximately 17,000 new cases in 2007 [13]. The prevalence of HBV in Canada and USA was also low and decreased among both sexes and across all ages between 1990 and 2005 due to wide-spread vaccination. Recent study indicated deceleration in the incidence of HCC around 2006 in USA and there was no increase in incidence rates since 2009 [14].

The long-term survival for patients with HCC remained poor despite the increasing median survival rate. The 6-month RSRs of HCC patients increased from 31.0% to 42.9% to 57.2% with a greater increase in the last two decades which could be attributed to the accelerated improvement of clinical management of HCC. But the long-term prognosis was poor illustrated by the facts that overall 5-year survival rates of patients with HCC were 4.7%, 10.6% and 18.2% respectively, highlighting the urgency of developing novel treatments to handle recurrence. Survival improvement across three decades could be attributed to the development of diagnosis and therapies including RFA, novel agents for TACE and surgical improvement in liver transplantation, and so on. Among them, RFA, already categorized as a curative treatment according to BCLC, showed similar clinical outcomes in managing smaller HCC compared with surgical resection [15, 16]. Recently, many molecular-targeted agents have been tried to improve HCC survival, such as Erlotinib targeting EGFR, RAD001 and CCI-779 targeting PI3K/AKT/MTOR, Lapatinib targeting HER2 and so on, however, only Sorafenib, which has multiple molecular targets, was approved by FDA in treating HCC [17–21].

Sexually speaking, males showed higher incidence of HCC compared with females and more importantly the gap between sexes in HCC incidence kept widening among three decades. Sex hormone induces the gender disparity in HBV virology and pathogenesis which indirectly contributes to the sexual difference of HCC incidence [22]. The widening disparities of incidence between sexes can be arisen from higher HBsAg positivity and higher alcohol and cigarette consumption in males [23]. In addition, recent study showed that estrogen suppresses HCC cell growth through upregulation of NLRP3 inflammasome [24]. However, there is an increasingly urgent need for research to identify the genetic difference and other risk factors responsible for the difference in the incidence between sexes. We demonstrated that the survival rates for both males and females kept increasing during three decades. Females showed significant advantage in 6-month survival in first decade and this advantage diminished in the following decades indicating the improvement of treatment and easy access of medical resources narrowed the biological gap between sexes. More importantly, after being stratified by age and decade, females showed significantly higher 6-month RSR compared with males in most cases, except for age group over 70 in 1993-2002 and 2003-2012, and this advantage in females was more evident in younger population. In addition, the narrowing survival gap between sexes can be seen in 12-month and 24-month RSR in most cases except the group aged over 70 in the last two decades, and this trend was also illustrated by Kaplan-Meier curves. This age-dependent survival superiority as well as lower incidence of HCC in females indicated the protective role of estrogen in HCC [24].

Racially, Others showed the highest HCC incidence, and the incidence difference between Blacks and Others narrowed in the last two decades due to the more rapidly increase rate of incidence in Blacks in that period. Others contained Asian population in which high incidence of HCC can be found. Our data also demonstrated that Whites had higher 6-month, 12-month and 24-month RSRs compared with Blacks. Recent study showed that no significant differences in tumor size, grade, or overall clinical stage were found among races; however, lower surgery rate was found in Blacks. In addition, Blacks showed the longest delay to surgery [25]. Taken together, this study highlights social and cultural factor in the management of HCC and therefore there is an urgent need to improve the surgery rate in Blacks.

In respect of different SES groups, incidence varied in three SES groups, with the highest incidence for medium-poverty group whereas high-poverty and low-poverty groups share similarly lower incidence. In addition, lower poverty was related with higher survival rate and the survival difference among three SES groups kept widening in time order. And significant survival advantage of low-poverty group can only be seen in the third decade. These may be due to the fact that patients in high-poverty group were kept away from better medical resources financially. The SES disparity between Whites and Blacks may contribute to the parallel changes of incidence and survival between them. Since the inferiority in finance and the longer delay between diagnosis and surgery of Blacks kept them away from better medical consultation and possibility of treating HCC in early stage.

Cox regression analysis for 1983-2012 indicated that sex, age, race and SES are independent predictors for prognosis. More importantly, Cox regression analysis for three decades showed that SES became increasingly significantly related with the survival time, with ever-decreasing *p* values of *p* = 0.027, *p* = 0.001 and *p*< 0.001 for each decade respectively. With more and more novel and effective treatments emerging with time, SES played an increasingly important role in determining whether HCC patients can access more suitable treatment in time. Similar tendency can be proved by Kaplan-Meier survival analysis with ever-decreasing *p* values of *p* = 0.3062, *p* = 0.0120 and *p*< 0.001 in three decades respectively (Figure 4d). Interestingly, after stratification of patients by age, race was significantly related with the survival time of HCC patients even in the age group in which SES was not significantly associated with the survival time. Taken together, although the SES distribution between races may be partly accountable for the similar survival tendencies between races and SES groups, risk factors like biological difference and lifestyle may contribute a considerable part in survival time and thereby further researches are needed to clarify real racial difference in survival.

It is noteworthy that we calculated the incidence, survival data and their tendencies over three decades by a mass patient population from SEER database, but this study was limited as the result shown can only demonstrate the trends in specific SEER regions and should be interpreted with cautions while being applied in other regions. Moreover this study may be affected by the under-registration, misclassification and variation of SES within and among counties [26].

In general, the incidence of HCC kept increasing in three decades partly due to the increasing prevalence of NAFLD whose risk factors are believed to be obesity and T2DM [27]. Therefore this study predicted the effectiveness of preventing HCC by additional control of obesity and T2DM in developing countries in which the prevalence of obesity and T2DM kept increasing whereas infection rate of HBV and HCV remained high. In addition, a modest survival improvement in HCC patients was also confirmed but the long-term prognosis was gloomy. Analyzing the survival of HCC in the past three decades showed us the shifts of clinical outcomes of HCC, and more importantly highlighted the urgency of improving surgery rate of Blacks. Furthermore, the study may help predict the future tendency of incidence and survival. This study may help better design healthcare policies and clinical management programs to balance the survival disparities between SES groups, races, ages and sexes confirmed in this study and thereby improve the clinical management of HCC.

## MATERIALS AND METHODS

### HCC cases from SEER program

All data of patients with HCC between 1983 and 2012 were accessed from the SEER program of the National Cancer Institute. And we collected incidence data from the original nine SEER sites and survival data from eighteen SEER sites respectively.

### Categorized HCC cases over the course of three decades

SEER*Stat version 8.3.2 was used for all data collection, incidence and survival analysis. Patient inclusion criteria based on the Site recode International classification of Diseases for Oncology, third Edition 2008 Liver (C22.0), and year of diagnosis: 1983-2012. HCC are histologically defined by the following International Classification of Diseases for Oncology, third Edition histology codes for malignant cases: hepatocellular carcinoma (8170/2, 8170/3, 8171/3, 8172/3, 8173/3, 8174/3, 8175/3). We analyzed the incidence and RSR data between 1983 and 2012 after being divided by three decades. In addition, patients were stratified by sex, SES, race and age. We excluded the cases of HCC diagnosed by autopsy or reported only on a death certificate. We preformed defining different SES groups as described previously, briefly SES was defined by the county poverty rate that were categorized into three levels [28].

### Incidence and survival data analysis

Incidence and RSR data were obtained from SEER*Stat version 8.3.2 and processed as previously described. Briefly incidence data were calculated per 100,000 persons and RSR refers to deaths attributed to NSCLC directly [28]. Kaplan-Meier curves were constructed by GraphPad Prism 6.0 and differences between the curves were assessed by a two-tailed log-rank test. A two-tailed *p* value < 0.01 was believed to be statistically significant.

### Cox regression analyses and correlation analysis

In Cox regression analysis and correlation analysis, we only included the cases of Whites and Blacks due to the heterogeneity in racial composition of Others. In addition, the original high-poverty group and medium-poverty group were redefined as “med-high-poverty group”. Spearman's rank correlation analysis was performed because the variant race was not normally distributed. StataMP 14 was used for Cox regression analyses and Spearman rank correlation analysis. A two-tailed *p* value < 0.01 was considered to be statistically significant.

## SUPPLEMENTARY FIGURES AND TABLES




