# Supplementary material for: Improved survival of patients with hepatocellular carcinoma and disparities by age, race, and socioeconomic status by decade, 1983–2012
Source: Oncotarget. 2016 Jul 29;7(37):59820–33. doi: 10.18632/oncotarget.10930 (PMC5312351; doi:10.18632/oncotarget.10930)
Supplement: Supplementary file 1 [file oncotarget-07-59820-s001.pdf]

Improved survival of patients with hepatocellular carcinoma and disparities by age, race, and socioeconomic status by decade, 1983–2012

SUPPLEMENTARY FIGURES AND TABLES

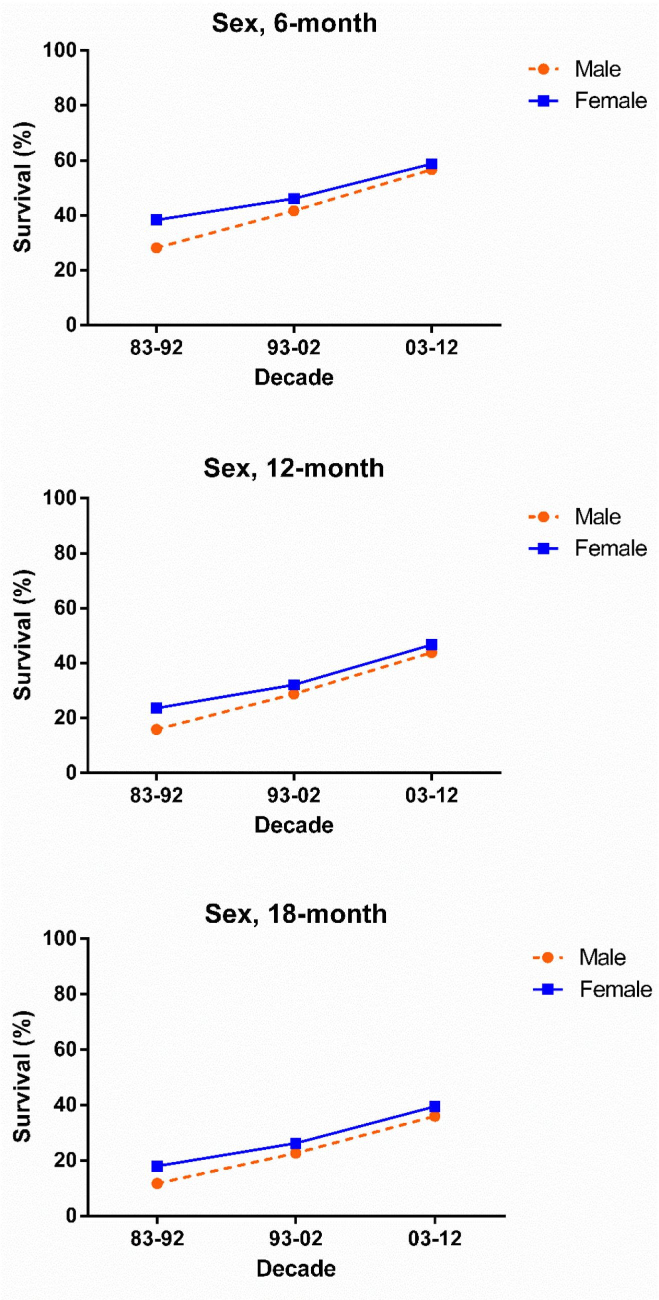

Supplementary Figure S1: 6-month, 12-month, and 18-month relative survival rates for male (orange) and female (blue) with HCC from 1983 to 2012 at eighteen SEER sites and calendar period.

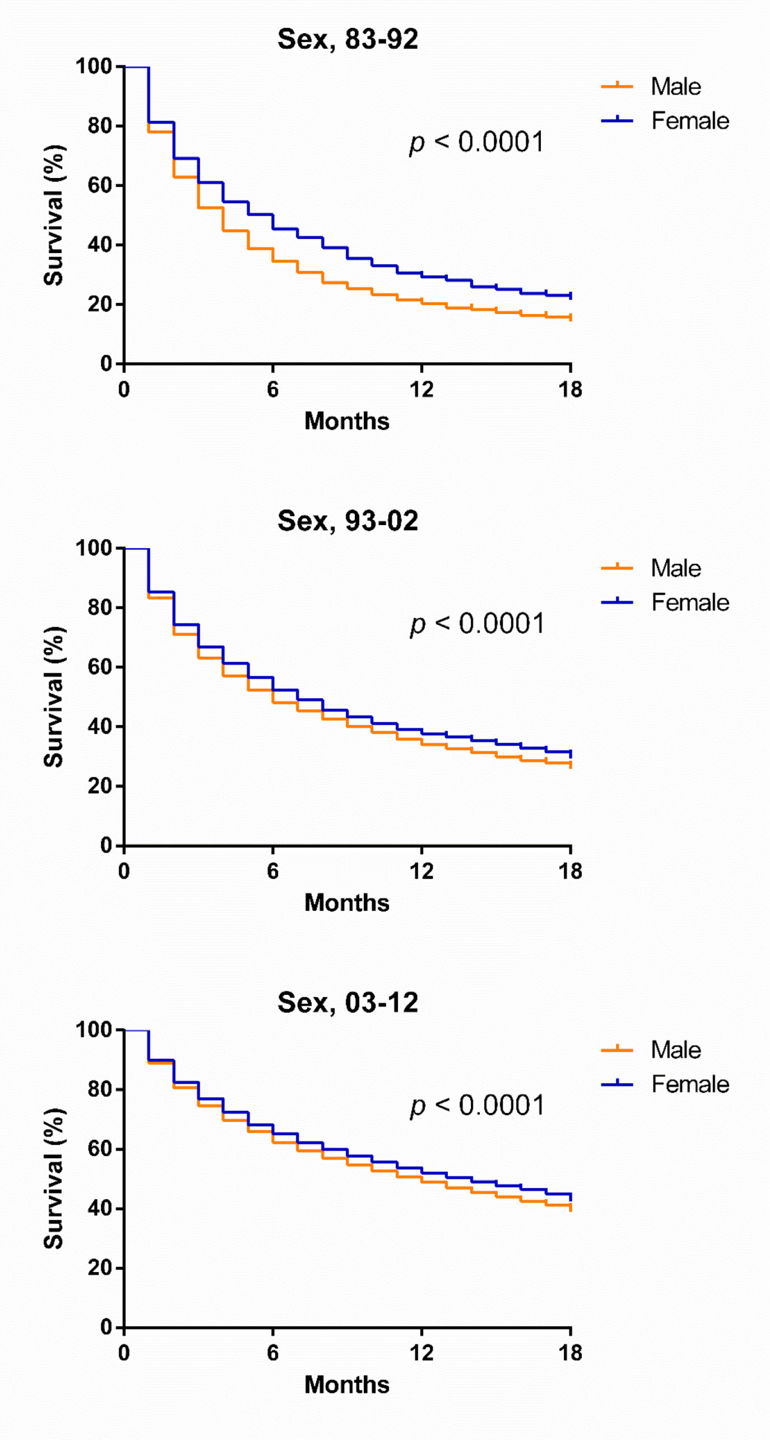

Supplementary Figure S2: Kaplan-Meier survival analysis for male (orange) and female (blue) with HCC from eighteen SEER sites from 1983 to 2012 according to calendar period.

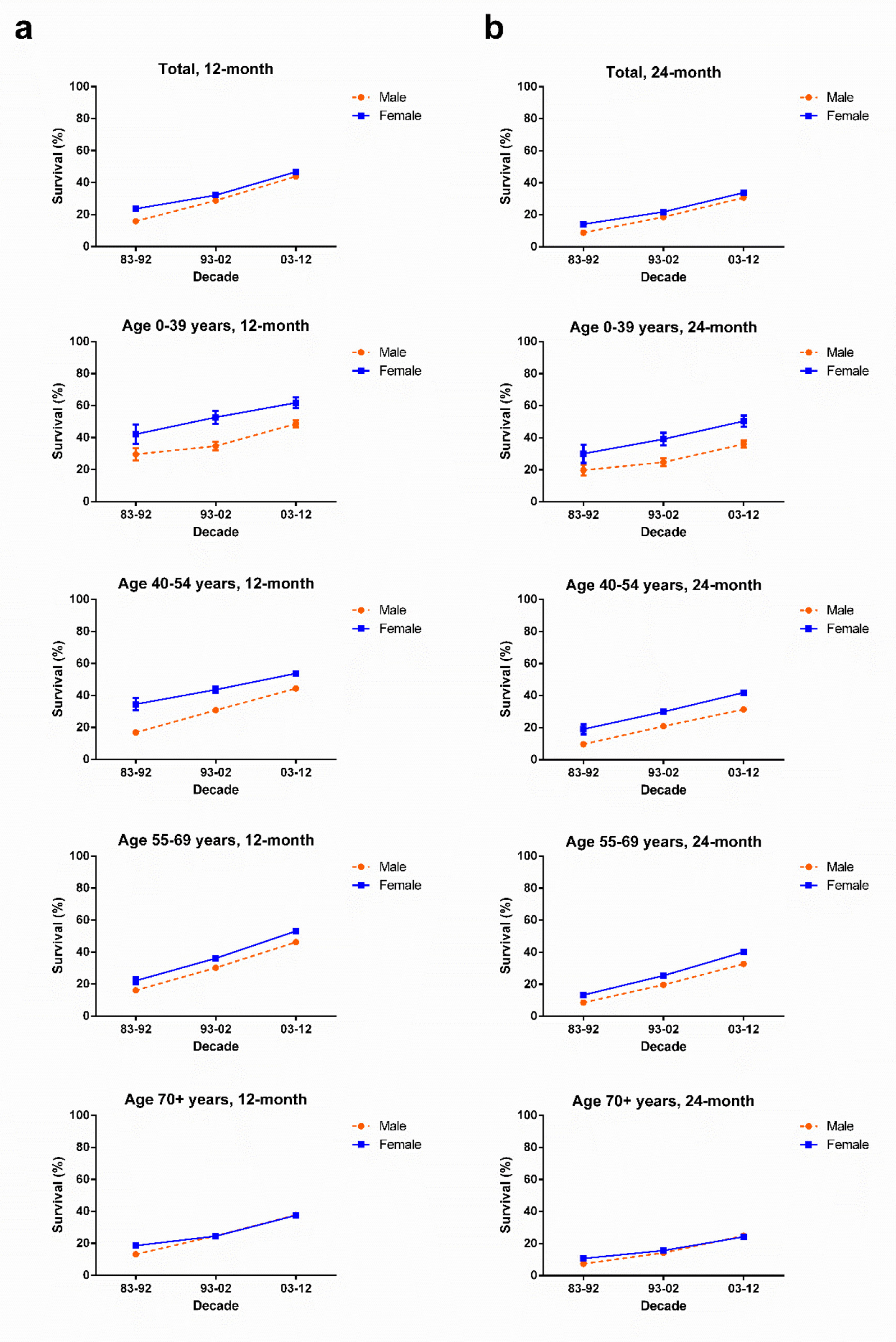

Supplementary Figure S3: 12 a. and 24-month b. relative survival rates for male (orange) and female (blue) with HCC at eighteen SEER sites from 1983 to 2012 according to age group (total and ages 0-39, 40-54, 55-69, and 70+ years).

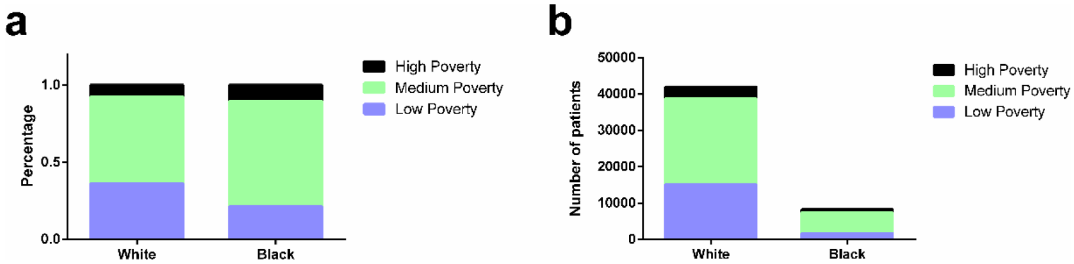

Supplementary Figure S4: Distribution of SES by race for patients with HCC at eighteen SEER sites from 1983 to 2012. Percentage **a.** and number **b.** of patients with HCC in low-poverty, medium-poverty, and high-poverty groups.

Supplementary Table S1: The incidence of HCC according to age group and decade within sex, SES, and race groups from 1983 to 2012 at the nine original SEER sites. Data are incidence per 100,000 people by year of diagnosis, with the number of patients in parentheses

See Supplementary File 1

Supplementary Table S2: 12-month and 24-month relative survival rates of HCC patients according to sex, age group, and calendar period from 1983 to 2012 at eighteen SEER sites

Decade	Age Group	Sex	
		Male	Female
83-92	12-Mo RSR		
	All	15.9 ± 0.7 (3145)	23.6 ± 1.2 (1188)***
	0-39	29.5 ± 3.8 (144)	42.1 ± 6.1 (67)***
	40-54	16.9 ± 1.7 (514)	34.5 ± 3.8 (160)***
	55-69	16.1 ± 1.0 (1416)	22.1 ± 2.1 (409)***
	70+	13.2 ± 1.1 (1071)	18.6 ± 1.7 (552)***
	24-Mo RSR		
	All	8.8 ± 0.5	14.1 ± 1.0***
	0-39	19.7 ± 3.3	30.0 ± 5.8***
	40-54	9.6 ± 1.3	19.0 ± 3.1***
	55-69	8.5 ± 0.8	13.3 ± 1.7***
	70+	7.4 ± 0.9	10.7 ± 1.4***
93-02	12-Mo RSR		
	All	28.8 ± 0.4 (11637)	32.1 ± 0.7 (4168)***
	0-39	34.7 ± 2.6 (255)	52.7 ± 4.1 (150)***
	40-54	30.8 ± 0.8 (3224)	43.6 ± 2.1 (587)***
	55-69	30.2 ± 0.7 (4493)	36.1 ± 1.3 (1397)***
	70+	24.7 ± 0.8 (3565)	24.5 ± 1.0 (2034)***
	24-Mo RSR		
	All	18.5 ± 0.4	21.8 ± 0.7***
	0-39	24.7 ± 2.3	39.1 ± 4.0***
	40-54	20.9 ± 0.7	29.9 ± 1.9***
	55-69	19.6 ± 0.6	25.4 ± 1.2***
	70+	14.2 ± 0.6	15.6 ± 0.9***
03-12	12-Mo RSR		
	All	43.9 ± 0.3 (33662)	46.7 ± 0.5 (9840)***
	0-39	48.6 ± 2.1 (584)	61.8 ± 3.3 (227)***
	40-54	44.4 ± 0.6 (8506)	53.8 ± 1.3 (1525)***
	55-69	46.2 ± 0.4 (16911)	53.1 ± 0.8 (3852)***
	70+	37.8 ± 0.6 (7661)	37.5 ± 0.8 (4236)***
	24-Mo RSR		
	All	30.7 ± 0.3	33.8 ± 0.5***
	0-39	36.0 ± 2.1	50.4 ± 3.5***
	40-54	31.4 ± 0.5	41.9 ± 1.4***
	55-69	32.7 ± 0.4	40.1 ± 0.9***
	70+	24.9 ± 0.6	24.2 ± 0.7***

Data are means ± standard error of the mean, with number of patients in parentheses.

Abbreviations: Mo, month; RS, relative survival; SEM, standard error of the mean.
*$p < 0.01$, **$p < 0.001$, and ***$p < 0.0001$ for comparisons with the Male group.

Supplementary Table S3: Summary data for Cox regression analysis of survival in patients with HCC in different age groups from 1983 to 2012 at eighteen SEER sites

Variable	Hazard Ratio (95% CI)	*p*-value
**Age 0-39 years**		
Univariate		
Sex	0.799 (0.686 − 0.932)	< 0.001
Age	1.026 (1.017 -1.034)	< 0.001
Race	1.541 (1.311 − 1.812)	< 0.001
SES	1.267 (1.086 − 1.477)	= 0.003
Multivariate		
Sex	0.823 (0.705 − 0.960)	= 0.013
Age	1.023 (1.015 -1.032)	< 0.001
Race	1.406 (1.193 − 1.656)	< 0.001
SES	1.192 (1.020 − 1.392)	= 0.027
**Age 40-54 years**		
Univariate		
Sex	0.793 (0.748 − 0.841)	< 0.001
Age	0.990 (0.985 − 0.996)	= 0.001
Race	1.341 (1.275 − 1.411)	< 0.001
SES	1.155 (1.104 − 1.207)	< 0.001
Multivariate		
Sex	0.782 (0.737 − 0.830)	< 0.001
Race	1.343 (1.276 − 1.413)	< 0.001
SES	1.129 (1.080 − 1.181)	< 0.001
**Age 55-69 years**		
Univariate		
Sex	0.843 (0.811 − 0.875)	< 0.001
Age	1.017 (1.014 − 1.021)	< 0.001
Race	1.200 (1.156 − 1.245)	< 0.001
SES	1.100 (1.065 − 1.135)	< 0.001
Multivariate		
Sex	0.825 (0.794 − 0.858)	< 0.001
Age	1.020 (1.016 − 1.023)	< 0.001
Race	1.203 (1.159 − 1.249)	< 0.001
SES	1.083 (1.050 − 1.118)	< 0.001
**Age 70+ years**		
Univariate		
Sex	0.970 (0.935 − 1.005)	= 0.096
Age	1.023 (1.020 − 1.026)	< 0.001
Race	1.067 (1.008 − 1.130)	= 0.026
SES	1.045 (1.009 − 1.083)	= 0.015

Abbreviations: 95% CI, 95% confidence interval; SES, socioeconomic status.

Supplementary Table S4: 12-month and 24-month relative survival rates of HCC patients according to race, age group, and calendar period from 1983 to 2012 at eighteen SEER sites

Decade	Age Group	Race		
		White	Black	Other
83-92	12-Mo RSR			
	All	18.2 ± 0.80 (2638)	13.0 ± 1.5 (534)	19.8 ± 1.2 (1155)
	0-39	55.4 ± 5.1 (97)	16.2 ± 6.6 (31)**	19.3 ± 4.3 (83)***
	40-54	23.3 ± 2.4 (306)	13.6 ± 3.3 (111)	21.0 ± 2.6 (255)
	55-69	17.4 ± 1.1 (1112)	13.3 ± 2.3 (232)	19.9 ± 1.8 (478)
	70+	14.4 ± 1.1 (1123)	11.4 ± 2.6 (160)	19.1 ± 2.2 (339)
	24-Mo RSR			
	All	10.3 ± 0.6	6.8 ± 1.1	11.8 ± 1.0
	0-39	38.7 ± 5	9.7 ± 5.3	13.3 ± 3.7**
	40-54	13.7 ± 2	5.5 ± 2.2	12 ± 2.1
	55-69	9.6 ± 0.9	7.3 ± 1.8	10.6 ± 1.4
	70+	7.5 ± 0.8	6.3 ± 2.1	12.9 ± 1.9
93-02	12-Mo RSR			
	All	28.3 ± 0.5 (9765)	23.7 ± 1.0 (1806)**	35.3 ± 0.8 (4183)***
	0-39	49.2 ± 3.4 (221)	28.6 ± 5.4 (74)	34.2 ± 3.4 (207)
	40-54	34.1 ± 1.0 (2196)	23.7 ± 1.7 (612)***	35.4 ± 1.5 (988)
	55-69	29.7 ± 0.8 (3493)	25.8 ± 1.7 (704)	37.7 ± 1.2 (1670)***
	70+	22.6 ± 0.7 (3855)	19.2 ± 2 (416)	32.3 ± 1.3 (1318)***
	24-Mo RSR			
	All	18.2 ± 0.4	13.6 ± 0.8***	24.6 ± 0.7***
	0-39	35.4 ± 3.2	18.6 ± 4.7	26.0 ± 3.1
	40-54	23.8 ± 0.9	13.3 ± 1.4***	24.4 ± 1.4
	55-69	19.1 ± 0.7	15.4 ± 1.4	27.1 ± 1.1***
	70+	13.0 ± 0.6	10.0 ± 1.6	21.3 ± 1.2***
03-12	12-Mo RSR			
	All	44.0 ± 0.3 (29121)	38.4 ± 0.7 (5840)***	50.4 ± 0.6 (8348)***
	0-39	63.8 ± 2.5 (388)	40.9 ± 4.2 (156)***	42.4 ± 3.1 (263)***
	40-54	46.8 ± 0.6 (6725)	36.7 ± 1.3 (1448)***	48.9 ± 1.2 (1816)
	55-69	47.6 ± 0.4 (13848)	40.2 ± 0.9 (3361)***	54.1 ± 0.9 (3448)***
	70+	34.6 ± 0.6 (8160)	33.8 ± 1.7 (875)	47.6 ± 1.0 (2821)***
	24-Mo RSR			
	All	30.4 ± 0.3	25.8 ± 0.6***	38.4 ± 0.6***
	0-39	47.7 ± 2.7	33.9 ± 4.1	32.5 ± 3.0**
	40-54	33.5 ± 0.6	24.1 ± 1.2***	37.7 ± 1.2
	55-69	33.6 ± 0.4	27.3 ± 0.9***	42.1 ± 0.9***
	70+	21.3 ± 0.5	21.4 ± 1.6	34.9 ± 1.0***

Data are means ± standard error of the mean, with number of patients in parentheses.

Abbreviations: Mo, month; RS, relative survival; SEM, standard error of the mean.
*$p < 0.01$, **$p < 0.001$, and ***$p < 0.0001$ for comparisons with the White group.

Supplementary Table S5: 12-month and 18-month relative survival rates of HCC patients according to SES, age group, and calendar period from 1983 to 2012 at eighteen SEER sites

Decade	Age Group	SES		
		Low Poverty	Medium Poverty	High Poverty
83-92	12-Mo RSR			
	All	18.9 ± 0.9 (1827)	17.5 ± 0.8 (2435)	14.8 ± 4.3 (71)
	0-39	42.6 ± 5.5 (80)	30.9 ± 4.1 (131)	0.0 ± 0.0 (0)
	40-54	27.3 ± 2.7 (266)	17.2 ± 1.9 (403)	0.0 ± 0.0 (5)
	55-69	16.8 ± 1.4 (754)	18.1 ± 1.2 (1042)	10.5 ± 5.7 (29)
	70+	15.1 ± 1.4 (727)	14.8 ± 1.3 (859)	20.2 ± 6.9 (37)
	24-Mo RSR			
	All	10.6 ± 0.7	10.0 ± 0.6	12.0 ± 4.0
	0-39	30.1 ± 5.1	20.9 ± 3.6	0.0 ± 0.0
	40-54	14.7 ± 2.2	10.1 ± 1.5	0.0 ± 0.0
	55-69	8.7 ± 1.0	10.4 ± 1.0	3.5 ± 3.4
	70+	9.0 ± 1.1	7.6 ± 1.0	20.2 ± 6.9
93-02	12-Mo RSR			
	All	31.2 ± 0.7 (5236)	29.0 ± 0.5 (9772)	28.6 ± 1.6 (795)
	0-39	51.8 ± 3.8 (181)	33.0 ± 2.7 (302)***	43.4 ± 10.8 (22)
	40-54	35.8 ± 1.4 (1232)	31.1 ± 1.0 (2360)***	33.6 ± 3.2 (218)***
	55-69	34.7 ± 1.1 (1881)	30.3 ± 0.8 (3741)***	27.7 ± 2.8 (268)***
	70+	22.8 ± 1.0 (1942)	25.7 ± 0.8 (3369)***	24.5 ± 2.6 (287)***
	24-Mo RSR			
	All	21.3 ± 0.6	18.5 ± 0.4***	18.1 ± 1.4***
	0-39	37.3 ± 3.7	24.1 ± 2.5***	29.0 ± 9.9***
	40-54	24.7 ± 1.2	20.7 ± 0.8***	24.8 ± 3.0
	55-69	24.3 ± 1.0	19.5 ± 0.7***	18.1 ± 2.4***
	70+	14.5 ± 0.9	15.0 ± 0.7***	11.8 ± 2.0***
03-12	12-Mo RSR			
	All	48.3 ± 0.4 (14229)	43.8 ± 0.3 (25673)***	35.3 ± 0.8 (3594)***
	0-39	57.9 ± 3.0 (279)	49.4 ± 2.4 (457)***	48.2 ± 6.0 (74)***
	40-54	50.6 ± 0.9 (3206)	44.8 ± 0.7 (5882)***	35.8 ± 1.6 (941)***
	55-69	51.6 ± 0.6 (6677)	46.8 ± 0.5 (12408)***	36.8 ± 1.2 (1676)***
	70+	40.3 ± 0.8 (4067)	37.1 ± 0.6 (6926)***	30.7 ± 1.7 (903)***
	24-Mo RSR			
	All	35.3 ± 0.4	30.4 ± 0.3***	22.7 ± 0.8***
	0-39	44.9 ± 3.2	38.2 ± 2.4***	32.9 ± 5.7***
	40-54	38.2 ± 0.9	31.5 ± 0.6***	24.3 ± 1.5***
	55-69	37.8 ± 0.7	33.3 ± 0.5***	24.5 ± 1.2***
	70+	27.9 ± 0.8	23.8 ± 0.6***	16.6 ± 1.4***

Data are means ± standard error of the mean, with number of patients in parenthesis.

Abbreviations: Mo, month; RS, relative survival; SEM, standard error of the mean.
*$p < 0.01$, **$p < 0.001$, and ***$p < 0.0001$ for comparisons with the Low Poverty group.

Supplementary Table S6: Summary data for race distribution by SES and calendar period in patients with HCC from 1983 to 2012 at eighteen SEER sites

Period	SES	Number	Race		
			White	Black	Other
1983-2012					
	Total	64095	65.5%	12.9%	21.5%
	Low Poverty	21482	70.7%	8.1%	21.2%
	Medium Poverty	38143	61.8%	14.9%	23.4%
	High Poverty	4470	72.9%	19.4%	7.7%
1983-1992					
	Total	4364	60.9%	12.4%	26.6%
	Low Poverty	1846	81.2%	6.1%	12.7%
	Medium Poverty	2447	45.2%	17.6%	37.2%
	High Poverty	71	76.1%	1.4%	22.5%
1993-2002					
	Total	15881	62.0%	11.5%	26.5%
	Low Poverty	5258	69.9%	6.8%	23.4%
	Medium Poverty	9826	56.8%	13.7%	29.5%
	High Poverty	797	75.0%	14.6%	10.4%
2003-2012					
	Total	43850	67.3%	13.5%	19.2%
	Low Poverty	14378	69.7%	8.9%	21.4%
	Medium Poverty	25870	65.2%	15.0%	19.7%
	High Poverty	3602	72.3%	20.8%	6.9%
