# Supplementary material for: Improved survival of patients with hepatocellular carcinoma and disparities by age, race, and socioeconomic status by decade, 1983–2012
Source: Oncotarget. 2016 Jul 29;7(37):59820–33. doi: 10.18632/oncotarget.10930 (PMC5312351; doi:10.18632/oncotarget.10930)
Supplement: Supplementary file 2 [file oncotarget-07-59820-s002.docx]

**Supplementary Table S1**. The incidence of HCC according to age group and decade within sex, SES, and race groups from 1983 to 2012 at the nine original SEER sites. Data are incidence per 100,000 people by year of diagnosis, with the number of patients in parentheses.

|  | | **Age**  **Group** | **Decade** | | |
| --- | --- | --- | --- | --- | --- |
| **Variable** | |  | **1983-1992** | **1993-2002** | **2003-2012** |
| Total |  |  |  |  |  |
|  |  | 0-85+ | 1.9 (4057) | 3.1 (7506) | 4.9 (14972) |
|  |  | 0-39 | 0.1 (216) | 0.2 (257) | 0.2 (339) |
|  |  | 40-54 | 1.7 (630) | 3.3 (1775) | 5.2 (3423) |
|  |  | 55-69 | 5.9 (1703) | 9.2 (2727) | 17.4 (7188) |
|  |  | 70+ | 8.5 (1508) | 12.5 (2747) | 17.0 (4022) |
| Sex | Male |  |  |  |  |
|  |  | 0-85+ | 3.2 (2946) | 5.0 (5535) | 8.1 (11583) |
|  |  | 0-39 | 0.2 (146) | 0.2 (173) | 0.3 (261) |
|  |  | 40-54 | 2.6 (477) | 5.6 (1482) | 8.8 (2876) |
|  |  | 55-69 | 9.8 (1320) | 15.1 (2109) | 29.6 (5889) |
|  |  | 70+ | 14.6 (1003) | 20.1 (1771) | 25.9 (2557) |
|  | Female |  |  |  |  |
|  |  | 0-85+ | 1.0 (1111) | 1.5 (1971) | 2.1 (3389) |
|  |  | 0-39 | 0.1 (70) | 0.1 (84) | 0.1 (78) |
|  |  | 40-54 | 0.8 (153) | 1.1 (293) | 1.6 (547) |
|  |  | 55-69 | 2.5 (383) | 4.0 (618) | 6.1 (1299) |
|  |  | 70+ | 4.6 (505) | 7.4 (976) | 10.4 (1465) |
| Race | white |  |  |  |  |
|  |  | 0-85+ | 1.4 (2485) | 2.3 (4543) | 3.8 (9215) |
|  |  | 0-39 | 0.1 (104) | 0.1 (111) | 0.1 (161) |
|  |  | 40-54 | 0.9 (285) | 2.2 (958) | 4.0 (2063) |
|  |  | 55-69 | 4.2 (1049) | 6.4 (1555) | 13.6 (4440) |
|  |  | 70+ | 6.7 (1047) | 10.3 (1919) | 13.1 (2551) |
|  | Black |  |  |  |  |
|  |  | 0-85+ | 3.2 (515) | 4.5 (961) | 7.7 (2364) |
|  |  | 0-39 | 0.2 (32) | 0.2 (41) | 0.3 (64) |
|  |  | 40-54 | 3.1 (108) | 6.1 (336) | 7.6 (584) |
|  |  | 55-69 | 9.8 (223) | 14.6 (374) | 33.5 (1374) |
|  |  | 70+ | 12.9 (152) | 14.0 (210) | 19.4 (342) |
|  | Other |  |  |  |  |
|  |  | 0-85+ | 7.4 (1052) | 8.7 (1970) | 10.1 (3343) |
|  |  | 0-39 | 0.6 (80) | 0.6 (103) | 0.5 (112) |
|  |  | 40-54 | 8.0 (235) | 8.8 (472) | 9.8 (768) |
|  |  | 55-69 | 20.5 (429) | 27.3 (782) | 29.6 (1348) |
|  |  | 70+ | 30.4 (308) | 34.5 (613) | 44.1 (1115) |
| SES | Low Poverty |  |  |  |  |
|  |  | 0-85+ | 1.6 (1805) | 2.6 (3597) | 4.5 (8098) |
|  |  | 0-39 | 0.1 (91) | 0.1 (124) | 0.2 (196) |
|  |  | 40-54 | 1.2 (258) | 2.6 (812) | 4.6 (1848) |
|  |  | 55-69 | 4.6 (743) | 7.5 (1264) | 15.4 (3793) |
|  |  | 70+ | 7.3 (713) | 11.1 (1397) | 15.9 (2261) |
|  | Medium Poverty |  |  |  |  |
|  |  | 0-85+ | 2.5 (2182) | 3.8 (3728) | 5.7 (6496) |
|  |  | 0-39 | 0.2 (125) | 0.2 (127) | 0.2 (133) |
|  |  | 40-54 | 2.4 (367) | 4.3 (925) | 6.0 (1479) |
|  |  | 55-69 | 7.6 (931) | 11.7 (1397) | 20.8 (3236) |
|  |  | 70+ | 10.0 (759) | 14.4 (1279) | 18.7 (1648) |
|  | High Poverty |  |  |  |  |
|  |  | 0-85+ | 1.5 (70) | 2.9 (180) | 4.6 (373) |
|  |  | 0-39 | 0.0 (0) | 0.1 (6) | 0.3 (10) |
|  |  | 40-54 | 0.6 (5) | 2.9 (38) | 5.7 (94) |
|  |  | 55-69 | 4.3 (29) | 7.9 (66) | 13.8 (157) |
|  |  | 70+ | 9.3 (36) | 13.1 (70) | 16.6 (112) |

Abbreviation: SES, socioeconomic status.
